# Limitations of ^18^F-FDG PET/CT in detecting direct bronchial metastasis from esophageal squamous cell carcinoma: A case report

**DOI:** 10.22038/aojnmb.2025.90754.1662

**Published:** 2026

**Authors:** Thong Huy Mai, Chau Quynh Anh, Ngo Thi Minh Hanh, Bui Quang Bieu, Khoa B Tran, Le Thi Thu Nga, Paolo Castellucci, Paeng Chul Jin, Mai Hong Son

**Affiliations:** 1 Department of Nuclear Medicine, 108 Military Central Hospital, Hanoi, Vietnam; 2 Faculty of Nuclear Medicine, Hanoi Medical University, Hanoi, Vietnam; 3 epartment of Histo-pathology, 108 Military Central Hospital, Hanoi, Vietnam; 4 Department of Radiation Oncology and Radiosurgery, 108 Military Central Hospital, Hanoi, Vietnam; 5 Cancer Institute, 108 Military Central Hospital, Hanoi, Vietnam; 6 Service of Nuclear Medicine, S. Orsola-Malpighi Hospital, University of Bologna, Bologna, Italy; 7 Department of Nuclear Medicine, Seoul National University Hospital, Seoul, Korea

**Keywords:** ^18^F-FDG PET/CT, Bronchial metastasis, Esophageal squamous cell carcinoma

## Abstract

Esophageal cancers predominantly metastasize through direct invasion, lymphatic dissemination, or hematogenous spread. Frequent metastatic locations comprise the liver, lymph nodes, lungs, and bones. However, direct bronchial metastases are extremely rare. We report a case of a 50-year-old male diagnosed with esophageal squamous cell carcinoma who received neoadjuvant chemoradiotherapy and subsequent esophagectomy with gastric pull-up reconstruction. Two years after surgery, follow-up imaging indicated suspicious bronchial lesion. On ^18^F-FDG PET/CT, the bronchial lesion exhibited only mild ^18^F-FDG uptake, which may underestimate its malignancy. Histopathological assessment verified metastatic squamous cell carcinoma, congruent with the primary esophageal tumor. This case illustrates the diagnostic limitations of ^18^F-FDG PET/CT in specific situations and emphasizes the necessity of incorporating clinical, endoscopic, and pathological findings in intricate cases.

## Introduction

 Esophageal squamous cell carcinoma (ESCC) is an aggressive malignancy known for its high propensity for both locoregional and distant metastases, most commonly affecting the lymph nodes, liver, lungs, and bones (1, 2). While pulmonary metastases from ESCC occur frequently, direct metastasis to the bronchial tree is exceptionally rare, with only sporadic cases reported in the literature (3). Such endobronchial involvement poses significant diagnostic challenges, as these lesions often lack distinctive clinical or imaging features and may be misinterpreted as primary bronchogenic tumors or other benign conditions (4). The clinical scenario becomes even more complex due to inherent limitations in current diagnostic modalities. Although ^18^F-FDG PET/CT has become invaluable in staging and detecting distant metastases of ESCC, the sensitivity for identifying small or atypically located metastases, including direct bronchial involvement, remains suboptimal. Factors such as lesion size, variable metabolic activity, and local physiological uptake can contribute to false-negative and false-positive results, some-times obscuring subtle malignant lesions and hindering accurate diagnosis. Therefore, histo-pathological confirmation is crucial for a definitive diagnosis in these atypical clinical scenarios (5). This case report illustrates a notably rare occurrence: isolated direct bronchial metastasis from ESCC, initially overlooked on ^18^F-FDG PET/CT imaging.

## Case Reports

**Figure 1:**
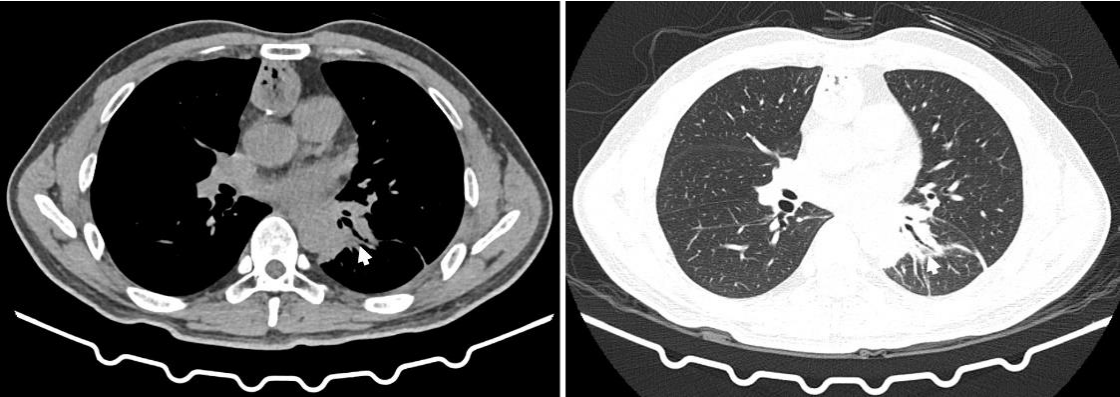
Axial non-contrast-enhanced CT images of the chest demonstrate a focal area of consolidation with surrounding ground-glass opacity (mediastinal window left panel, and lung window, right panel, **arrows**) in the left lower lobe, suggestive of either post-inflammatory change or malignant infiltration

**Figure 2:**
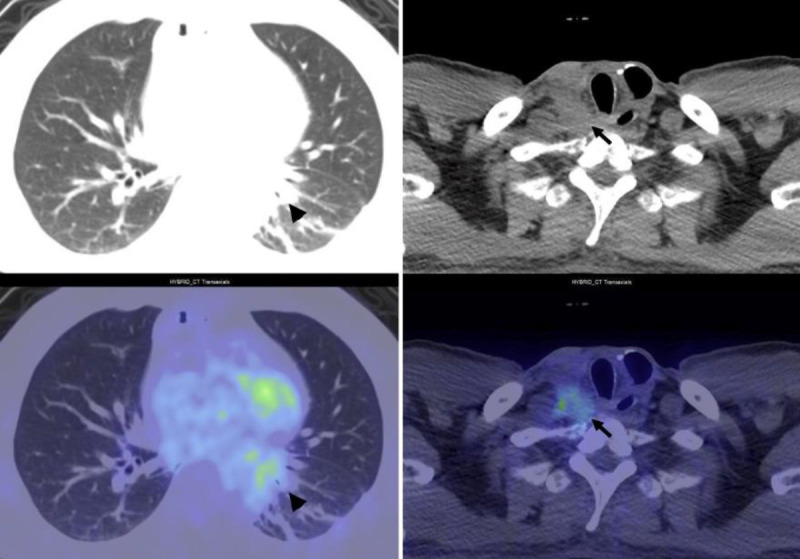
^18^F-FDG PET/CT fusion images demonstrate two distinct lesions: (**A**) A mildly ^18^F-FDG-avid lesion in the left lower lobe of the lung, infiltrating the left lower lobar bronchus with associated bronchial narrowing (**arrowheads**). (**B**) A hypermetabolic soft-tissue lesion in the right cervical region, adjacent to the upper trachea at the C7–T1 level, near the esophagogastric anastomosis (**arrows**)

 A 50-year-old male was diagnosed with ESCC, and an ^18^F-FDG PET/CT scan was performed in August 2022 for staging, revealing pT3N1M0. The patient subsequently received neoadjuvant chemoradiotherapy, followed by esophagectomy and gastric pull-up reconstruction in November 2022. After surgery, he was monitored with regular follow-up every three months, including contrast-enhanced chest and abdominal CT scans and upper endoscopy. No recurrence and or metastases were detected during this period. In March 2025, the patient presented with hoarseness and mild shortness of breath. A contrast-enhanced chest CT performed during routine follow-up revealed a focal consolidation area with surrounding ground-glass opacity in the left lower lobe (Figure 1). ^18^F-FDG PET/CT scan was performed for restaging. Two areas of concern were identified: a mildly ^18^F-FDG-avid lesion infiltrating the left lower lobar bronchus with associated bronchial narrowing (SUV_max_: 3.5) and another hypermetabolic soft-tissue lesion (SUV_max_: 4.1) adjacent to the right upper trachea at the level of C7–T1 vertebrae, near the esophagogastric anastomosis (Figure 2). Bronchoscopy revealed diffuse mucosal infiltration of the left lower bronchus. Biopsy specimens demonstrated clusters of malignant squamous cells with irregular hyperchromatic nuclei infiltrating fibrous stroma. 

**Figure 3:**
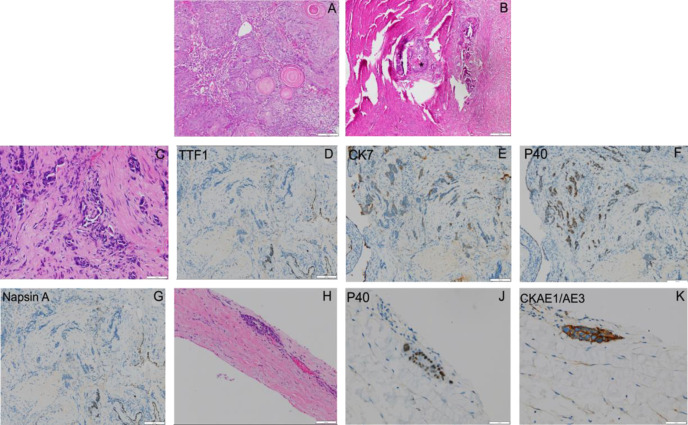
(**A**, **B**) Esophageal squamous cell carcinoma with keratin pearl formation and deep muscular invasion (H&E). (**C**) Lung biopsy showing malignant squamous cells infiltrating fibrous stroma (H&E, ×400). (**D**, **G**) Negative for TTF-1 and Napsin A. (**E**, **F**) Positive CK7 and P40 staining in bronchial lesion. (**H**, **J**, **K**) the hypermetabolic soft-tissue lesion (17 × 19 mm, SUV_max_ 4.1) adjacent to the right upper trachea (C7–T1 level), showing positivity for P40 and CKAE1/AE3

 Immunohistochemical staining was positive for CK7 and P40, and negative for Napsin A and TTF-1, consistent with squamous cell carcinoma (Figure 3A-E). Comparative review of prior histopathology confirmed similarity with the original esophageal tumor: keratinizing squamous cell carcinoma with keratin pearl formation in the pre-treatment biopsy, and deep invasion into the muscularis propria in the surgical specimen (Figure 3F–G). The multidisciplinary tumor board concluded that the bronchial lesion represented metastatic spread from the esophageal primary. 

**Figure 4:**
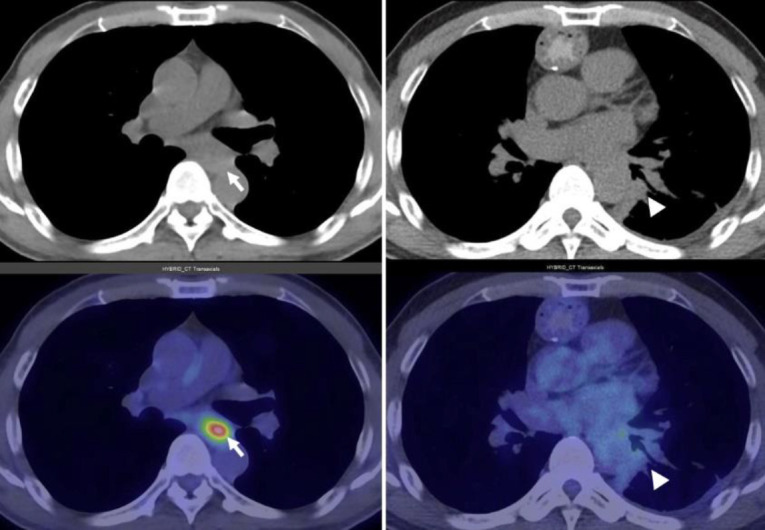
(**A**) Axial ^18^F-FDG PET/CT images from the initial staging scan (August 2022) demonstrate ^18^F-FDG-avid uptake in the mid-esophagus (**arrow**), corresponding to the primary tumor. (**B**) Follow-up ^18^F-FDG PET/CT performed in March 2025, after the patient had undergone esophagectomy with gastric pull-up reconstruction, shows a mildly hypermetabolic lesion in the left lower bronchus (**arrow head**), located adjacent to the previous tumor bed, suggestive of intraoperative tumor cell seeding

 Importantly, the anatomical location of the lesion near the original tumor bed suggests the possibility of intraoperative tumor cell seeding (Figure 4). The tracheal-side lesion was also biopsied and confirmed as metastatic squamous cell carcinoma (positive for P40 and CKAE1/AE3; Figure 3I–K). The patient was diagnosed with recurrent metastatic disease affecting the lower left bronchus and the right paratracheal lymph node. Systemic therapy was initiated with cisplatin, 5-fluorouracil, and pembrolizumab. After three cycles, the patient's voice and respiratory function improved, and his CT scan showed a reduction in the left lower lobe lung lesion as well as a decrease in the size of the right paratracheal lymph node. The patient was planned to complete three additional cycles of the regimen, followed by pembrolizumab consolidation for a total duration of two years.

## Discussion

 This case report describes a rare occurrence of direct bronchial metastasis stemming from esophageal squamous cell carcinoma, emphasizing significant diagnostic challenges, particularly regarding ^18^F-FDG PET/CT. Although ^18^F-FDG PET/CT is a well-established and essential tool for detecting recurrence and metastatic disease in esophageal cancer, the patient's case highlights its limitation in specific clinical scenarios.

 The most challenging diagnostic finding was the mild ^18^F-FDG uptake (SUV_max_: 3.5) detected in the left lower lobar bronchus. In a postoperative setting, this lower metabolic activity could be wrongly thought to be benign inflammatory responses, fibrotic changes, or primary lung cancer. Even though the lesion's infiltrative nature and mucosal involvement were apparent, these characteristics were inadequate for the differentiation between benign and malignant pathologies based only on imaging. Ultimately, bronchoscopy with histopathological confirmation was paramount in establishing the correct diagnosis. In this case, the infiltrative nature and mucosal involvement of the bronchial lesion made it difficult to distinguish from benign or malignant conditions on imaging. Diagnosis was made only by bronchoscopy and histopathological confirmation. Due to the close anatomical relationship between the bronchial lesion and the surgical site, surgical seeding or direct invasion by the primary tumor are possible explanations; however, this remains uncertain (6, 7). Though exceedingly rare, implantation 

metastasis should be considered in patients with unusual recurrence patterns following upper gastrointestinal surgery (8, 9). This case serves as a reminder that ^18^F-FDG PET/CT, though powerful, is not infallible. Clinicians must maintain a high index of suspicion and incorporate complementary diagnostic tools-especially endoscopic and pathological evaluation-to ensure accurate assessment and optimal patient management (10, 11).

## Conclusion

 Direct bronchial metastasis from esophageal squamous cell carcinoma is exceptionally uncommon and presents diagnostic difficulties. ^18^F-FDG PET/CT may underestimate the magnitude or characteristics of such lesions, particularly when ^18^F-FDG uptake is minimal. This case demonstrates the importance of correlating imaging findings with clinical, endoscopic, and histopathological information.
